# Failure of B Cell Tolerance in CVID

**DOI:** 10.3389/fimmu.2019.02881

**Published:** 2019-12-10

**Authors:** Christopher T. Richardson, Maria A. Slack, Gitika Dhillon, Carolina Z. Marcus, Jennifer Barnard, Arumugam Palanichamy, Ignacio Sanz, Richard John Looney, Jennifer H. Anolik

**Affiliations:** ^1^Department of Dermatology, University of Rochester Medical Center, Rochester, NY, United States; ^2^Division of Allergy, Immunology, and Rheumatology, Department of Medicine, University of Rochester Medical Center, Rochester, NY, United States; ^3^Division of Allergy and Immunology, Department of Pediatrics, University of Rochester Medical Center, Rochester, NY, United States; ^4^Division of Rheumatology, Department of Medicine, Lowance Center for Human Immunology, Emory University, Atlanta, GA, United States

**Keywords:** CVID, common variable immunodeficiency disorders, autoimmunity, B cell, B cell subpopulations, B cell tolerance, 9G4, VH4-34

## Abstract

Common variable immunodeficiency (CVID) comprises a group of related disorders defined by defects in B cell function and antibody production. Concurrent autoimmune features are common, but the underlying pathogenic mechanisms of autoimmunity in CVID are poorly understood. Overlap in some clinical and laboratory features suggests a shared pathogenesis, at least in part, with systemic lupus erythematosus (SLE). One important part of SLE pathogenesis is loss of B cell tolerance, an aspect that warrants further study in CVID. The study of inherently autoreactive 9G4^+^ B cells has led to a greater understanding of B cell tolerance defects in lupus. Study of these B cells in CVID has yielded conflicting results, largely due to differences in methodological approaches. In this study, we take a comprehensive look at 9G4^+^ B cells throughout B cell development in CVID patients and compare patients both with and without autoimmune features. Using flow cytometry to examine B cell subpopulations in detail, we show that only those CVID patients with autoimmune features demonstrate significant expansion of 9G4^+^ B cells, both in naïve and multiple memory populations. Examination of two autoreactive B cell subsets recently characterized in SLE, the activated naïve (aNAV) and double negative 2 (DN2) B cells, reveals an expanded 9G4^+^ DN2 population to be common among CVID patients. These results reveal that both multiple central and peripheral B cell tolerance defects are related to autoimmunity in CVID. Furthermore, these data suggest that the autoreactive DN2 B cell population, which has not previously been examined in CVID, may play an important role in the development of autoimmunity in patients with CVID.

## Introduction

Common variable immunodeficiency (CVID) is a heterogeneous group of antibody deficiency disorders. Approximately 25–30% of patients with CVID develop autoimmune disease (1, 2), which may be the presenting symptom in up to 17% of patients (3). Cytopenias are the most common autoimmune feature, but patients with CVID also develop a wide variety of other conditions of immune dysregulation including granulomatous lymphocytic interstitial lung disease, inflammatory bowel disease, celiac-like enteropathy, inflammatory arthritis, pernicious anemia, Sjogren syndrome, uveitis, vasculitis, thyroiditis, alopecia, vitiligo, hepatitis, primary biliary cirrhosis, sicca syndrome, and systemic lupus erythematosus (SLE) (4). Notably, these autoimmune and non-infectious complications confer a significantly increased mortality in CVID. Treatment efficacy is limited and remains non-specific given that the underlying pathogenesis of these complications remains undetermined.

CVID is characterized by a variety of defects in B cell function and antibody production (4), which has led to several classification schemes based on the flow cytometric characterization of B cell subpopulations (5–9). In each classification system, the observed B cell abnormalities correlate with the various non-infectious manifestations of CVID, including granulomatous disease, splenomegaly, and autoimmunity. These schemes highlight significant B cell defects in CVID including loss of isotype-switched memory B cells and plasma cells, expansion of transitional B cells and/or CD21lo B cells, and abnormal germinal center (GC) formation. The expansion of CD21lo B cells and abnormal GC function are of particular interest given these are shared features with SLE (10–12) and may help to explain the autoimmune features sometimes noted in CVID. Lack of CD21 on B cells has been noted on two recently-characterized and related B cell subsets that are increased in SLE, namely activated naïve (aNAV) (13) and double negative 2 (DN2) B cells (14). These subsets are thought to contribute to autoantibody formation and SLE pathogenesis via an extrafollicular (GC independent) activation pathway (14). The overlap of some B cell abnormalities with SLE and the success of B cell depletion therapy in CVID (15) suggest that a defect in B cell tolerance may play a significant role in the development of autoimmunity in CVID patients, though the precise mechanism and stage of tolerance loss remain unknown.

The generation of autoreactive B cells is inherent in the process of B cell development. Studies of monoclonal antibodies derived from single B cells in healthy individuals have shown that roughly 75% of newly formed B cells are self-reactive (16). As B cells develop in the bone marrow and mature in the periphery, this frequency of autoreactivity decreases through a series of tolerance checkpoints. While about 20% of naïve B cells in the peripheral blood remain self-reactive (16), only about 2% of IgM^+^ memory B cells normally exhibit autoreactivity (17). Failure of B cell tolerance at these checkpoints has been demonstrated in autoimmune diseases, such as systemic lupus erythematosus (SLE) and rheumatoid arthritis (18–20).

Further understanding of human B cell tolerance has been aided by the study of B cells that express a heavy chain variable region encoded by the VH4-34 gene. A unique hydrophobic patch on these heavy chains binds to self-antigens and can be detected with a monoclonal anti-idiotypic antibody called 9G4. Somatic mutation of this patch leads to loss of 9G4 reactivity, which directly correlates with loss of self-reactivity to particular autoantigens (21, 22). Therefore, 9G4 reactivity is a direct surrogate for autoreactivity and 9G4^+^ B cells are considered to be inherently autoreactive.

9G4^+^ B cells comprise roughly 5% of the total B cells in the peripheral blood of healthy individuals (10, 23–25). Peripheral tolerance mechanisms normally censor these B cells from entering germinal centers and becoming switched-memory B cells and plasma cells (10, 23), with serum titers of 9G4^+^ antibody virtually absent in healthy individuals (23, 25). In contrast, in SLE 9G4^+^ B cells are found at increased frequencies (10, 23), are readily found in germinal centers and memory B cell populations (10, 23, 26), and produce autoantibodies with serum titers that correlate with disease activity (10, 27–31). The expansion of 9G4^+^ B cells in memory populations is an indicator that peripheral B cell tolerance has been broken.

Although 9G4^+^ B cell expansion is most commonly found in SLE, there is some debate in the literature as to the potential expansion of 9G4^+^ B cells in CVID. While one group has reported an increase in 9G4^+^ B cells in the naïve and CD21lo B cell populations in a few patients with CVID type Ia (>20% CD21lo B cells) (32), another reports that VH4-34 transcripts were not increased among CD21lo B cells (33). In contrast, these transcripts appear to be increased in frequency in single IgG^+^ B cells from CVID patients with autoimmune cytopenia, as opposed to other CVID patients and healthy subjects (34). A more recent study using next generation sequencing of sorted naïve and switched memory B cells from CVID patients demonstrated an increased usage of VH4-34 in both the naïve and IGHG transcripts as compared to healthy controls (35). Comparison of these studies is limited by their use of different techniques to evaluate B cell subsets in different CVID populations with varying degrees of autoimmunity. In addition, one limitation to using VH4-34 transcripts as a surrogate for autoimmunity is that, while unmutated transcripts almost always correlate with autoreactive antibody, somatic hypermutation can result in loss of autoreactivity. In contrast, 9G4 reactivity directly correlates with self-reactivity, in particular to leukocytes and erythrocytes, and thus represents a better indicator of autoreactivity than the presence of VH4-34 transcripts (22). In this study, our analysis of 9G4^+^ B cells throughout peripheral B cell development demonstrates defects in early and late B cell tolerance checkpoints in CVID patients with autoimmunity.

## Materials and Methods

### Patient Selection and Sample Procurement

Eight CVID patients and twelve healthy controls were enrolled in this study. Detailed written informed consent was obtained from all patients and healthy donors in accordance with protocols approved by the Human Subjects Institutional Review Board of the University of Rochester Medical Center. All CVID patients met diagnostic criteria for CVID including marked decrease in serum IgG and either IgM or IgA (Table 1), lack of protective titers to vaccine antigens, the onset of clinical immunodeficiency at >2 years of age, and the exclusion of other defined causes of hypogammaglobulinemia. All patients were age 18 years or older. No patients received B cell depletion therapy or BAFF inhibitors.

**Table 1:** Patient demographics and immune status.

	**Gender**	**Age**	**IgG**	**IgA**	**IgM**	**Autoimmune Features**
**CVID (*****n*** **=** **4)**
1	F	45	Low	Low	Low	None
2	F	30	Low	Normal	Low	None
3	M	42	Low	Low	Low	None
4	M	77	Low	Low	Low	None
Total	2 (50)^*^	49 (30–77)^#^	4 (100)^$^	3 (75)^$^	4 (100)^$^	0 (0)^$^
**CVID-AI (*****n*** **=** **4)**
5	F	27	Low	Low	Low	Idiopathic thrombocytopenic purpura (ITP)
6	F	50	Low	Low	Low	Microscopic colitis, vitiligo
7	M	68	Low	Low	Low	ITP
8	F	55	Low	Low	Normal	Multiple sclerosis, Raynaud, lichen sclerosus
Total	3 (75)^*^	50 (27–68)^#^	4 (100)^$^	4 (100)^$^	3 (75)^$^	4 (100)^$^
**Healthy (*****n*** **=** **12)**
Total	7 (58)^*^	48 (32–77)^#^	ND	ND	ND	0 (0)^$^

*CVID, common variable immunodeficiency; CVID-AI, CVID with autoimmunity; ND, not done*.

* *Total females, n (%)*.

# *Age in years, average (range)*.

$ *Total low immunoglobulin, n (%)*.

### Cell Isolation and Flow Cytometry

Peripheral blood mononuclear cells (PBMC) from healthy controls and patients with CVID were isolated using Ficoll-Hypaque density gradient centrifugation (GE Healthcare). PBMCs were blocked with mouse and rat serum and stained at 4°C in buffer (1% bovine serum albumin in phosphate-buffered saline). The following anti-human antibodies were used in this study: anti-CD19 APC-Cy7, anti-IgD FITC, anti-CD3 Pacific Blue, anti-CD21 PE-Cy5, and anti-CD38 PE-Cy5.5 (BD Biosciences); anti-CD24 AlexaFluor 610 and anti-CD27 Qdot 605 (Invitrogen). Biotinylated anti-rat idiotypic 9G4 (kindly provided by Dr. Freda Stevenson, University of Southampton, Southampton, United Kingdom) was used in conjunction with streptavidin AlexaFluor 680 (Invitrogen). LIVE/DEAD Fixable Aqua Dead Cell Stain Kit (Invitrogen) was used for dead cell discrimination. Flow cytometric data were collected on an LSRII flow cytometer (BD Biosciences) and data analysis was performed with FlowJo software (TreeStar). B cell populations were analyzed as previously described by our group (36, 37). Flow cytometric analysis was performed without knowledge of the autoimmune status of the subject. The CVID or CVID-AI clinical status was assigned separately.

### Statistical Analysis

Statistical significance was assessed by the non-parametric Mann-Whitney test using GraphPad Prism (GraphPad Software). Differences were considered significant at *p* < 0.05. Error bars denote standard error of the mean.

## Results

### B Cell Abnormalities in CVID

**Figure 1:**
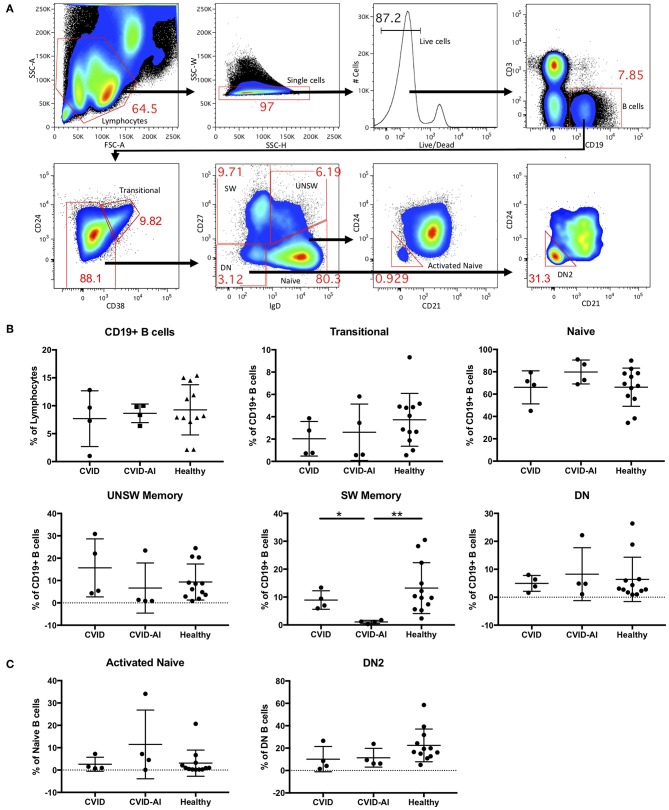
Analysis of B cell subsets in CVID patients with and without autoimmune features. **(A)** Flow cytometric analysis of total CD19^+^ B cells and seven B cell subsets: transitional (CD24^hi^ CD38^hi^), naïve (IgD^+^CD27^−^), class-switched memory (SW, IgD^−^CD27^+^), unswitched memory (UNSW, IgD^+^CD27^+^), double-negative (DN, IgD^−^CD27^−^), activated naïve (aNAV, IgD^+^CD27^−^CD21^−^CD24^−^), and double-negative 2 (DN2, IgD^−^CD27^−^CD21^−^CD24^−^). **(B,C)** As compared to both CVID and healthy subjects, the frequency of SW memory B cells in CVID-AI patients was decreased. No other differences in B cell subset frequencies were noted between healthy controls and CVID patients with or without autoimmune features. *p < 0.05, **p < 0.01.

Consistent with known defects in CVID, our cohort of eight patients exhibited levels of immunoglobulins below the normal range and a variety of autoimmune features (Table 1). For further analysis, CVID patients were divided into two groups, those with features of autoimmunity (CVID-AI) and those without (CVID). Total B cells and B cell subpopulations were analyzed by flow cytometry (Figure 1A).

No differences were found in the frequency of total B cells in the peripheral blood of these two CVID populations as compared to healthy controls (Figure 1B). We then analyzed the five major peripheral blood B cell subsets: transitional (CD24^hi^ CD38^hi^), naïve (IgD^+^CD27^−^), class-switched memory (SW, IgD^−^CD27^+^), unswitched memory (UNSW, IgD^+^CD27^+^), and double-negative (DN, IgD^−^CD27^−^). The frequency of SW memory B cells in CVID-AI was decreased as compared to both CVID patients without autoimmunity and healthy controls (Figure 1B). No other significant differences in B cell subset frequencies among CVID, CVID-AI, and healthy controls were found.

Given their role in lupus pathogenesis, we then evaluated the more recently characterized activated naïve (aNAV, IgD^+^CD27^−^CD21^−^CD24^−^) and double-negative 2 (DN2, IgD^−^CD27^−^CD21^−^CD24^−^) B cell populations. These B cell subsets have an activated phenotype, including loss of CD21 and CD24 (14). No differences in these B cell subsets were found among CVID, CVID-AI, and healthy controls (Figure 1C).

### Tolerance Defects in CVID

**Figure 2:**
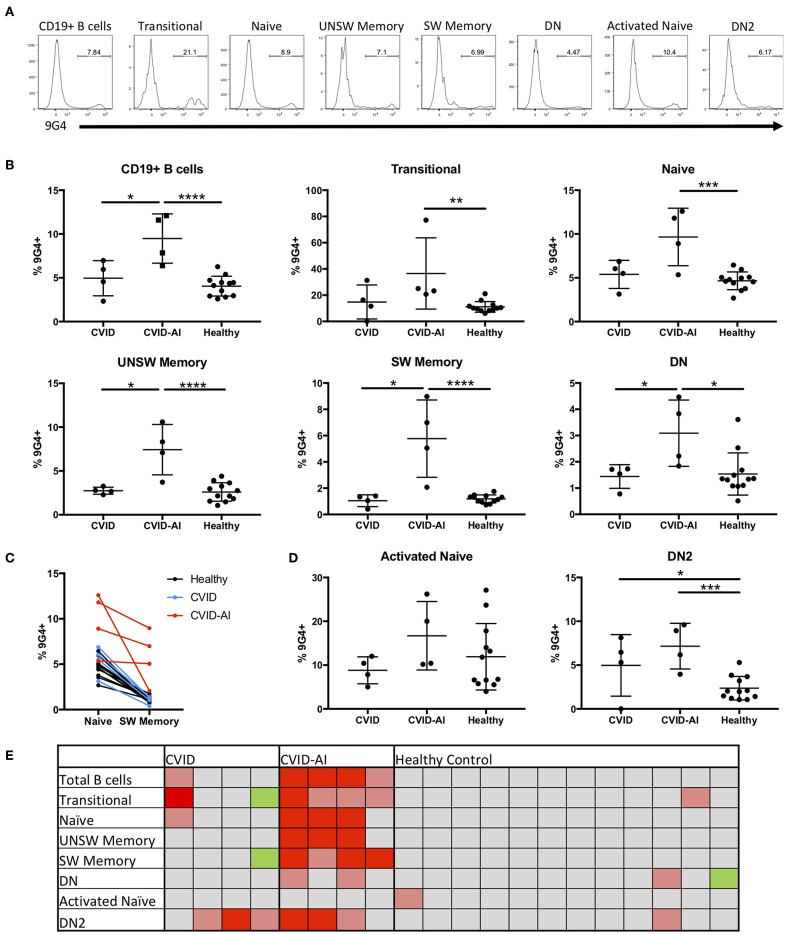
Expansion of 9G4^+^ B cells in CVID patients with autoimmune features. **(A)** Representative flow cytometric analysis of the frequency of 9G4^+^ B cells in one CVID-AI subject. **(B)** The frequency of 9G4^+^ B cells is increased in CVID-AI patients as compared to both CVID and healthy subjects. The frequency of 9G4^+^ B cells in all basic B cell subsets is increased in CVID-AI patients as compared to healthy controls, as well as in memory populations as compared to CVID. **(C)** The frequency of 9G4^+^ B cells decreases in the transition from naïve to SW memory B cells in both healthy (*p* < 0.0001) and CVID (*p* = 0.0051) subjects, but not in the CVID-AI group. **(D)** 9G4^+^ B cells are increased in the DN2 B cell subset for both CVID and CVID-AI, but not in activated naïve B cells. **(E)** Heatmap of the frequency of 9G4^+^ cells as compared to the mean of healthy controls: gray, within 2 standard deviations (SD); red, 4 SD above; pink, 2 SD above; green, 2 SD below. *p < 0.05, **p < 0.01, ***p < 0.001, ****p < 0.0001.

In order to evaluate potential defects in B cell tolerance in CVID patients, the frequency of 9G4^+^ B cells was evaluated throughout peripheral B cell development and maturation (Figure 2A). There was a significant expansion of 9G4^+^ B cells in CVID-AI patients as compared to healthy controls (Figure 2B). This expansion was near significance as compared to CVID subjects (*p* = 0.0571). CVID patients without autoimmune features did not exhibit this same expansion of 9G4^+^ B cells.

In order to evaluate at what point in B cell development and maturation this potential breach in tolerance occurs, the frequency of 9G4^+^ B cells was evaluated in the major B cell subsets in the peripheral blood. Consistent with known defects in SLE (10, 23), an expansion of 9G4^+^ B cells was observed in all B cell subsets in CVID-AI patients as compared to healthy controls (Figure 2B), including all memory B cell populations evaluated. Figure 2C highlights the change in the frequency of 9G4^+^ B cells during maturation from the naïve to SW memory B cell compartments. A significant decrease was observed in both healthy (*p* < 0.0001) and CVID (*p* = 0.0051) subjects (paired Student's *t*-test), but not for the CVID-AI group. In order to correlate the noted differences within and among individuals, a heatmap of the data was generated (Figure 2E). The mean of the healthy control population was used as a baseline, with 2- and 4-fold differences in standard deviation (SD) above and below the mean noted. This heatmap analysis corroborates the differences previously observed, but also highlights the variability among individuals within groups. The frequency of 9G4^+^ B cells was also evaluated in the aNAV and DN2 B cell populations (Figure 2D). While no increase was found in the aNAV population, there was an expansion of 9G4^+^ B cells in the DN2 population of CVID-AI patients.

## Discussion

The study of 9G4^+^ B cells has proven to be a powerful system to evaluate human B cell tolerance and autoreactivity. These inherently autoreactive B cells are found in all individuals, but outside of autoimmune states, are subject to tolerizing mechanisms that limit development, maturation, and antibody production. In this study, we demonstrate a loss of B cell tolerance in CVID patients with autoimmune features, with significantly increased frequencies of 9G4^+^ B cells throughout development, in contrast to CVID patients without autoimmune features and healthy controls.

B cells begin development in the bone marrow and are subject to central tolerance mechanisms that greatly reduce autoreactivity among the B cells that first enter the peripheral circulation, termed transitional B cells. Studies of these cells in autoimmune diseases, such as lupus have revealed defects in central tolerance (16). Defective central tolerance in lupus appears to be due to early interferon imprinting of immature B cells in the bone marrow that results in CD19 downregulation and impaired TLR9 responses (38). Our observation of an expansion of 9G4^+^ B cells in the transitional compartment of CVID patients with autoimmune features is suggestive of a central or early peripheral B cell tolerance defect. This fits well with data showing an increased interferon signature in CVID patients with inflammatory complications (39), as well as transcriptional evidence indicating possible defects in early B-cell development or selection against autoimmunity (35). Our data suggest that this defect in central tolerance, when present, appears to be largely censored in CVID patients without autoimmunity as evidenced by the relative lack of 9G4^+^ B cells in naïve and memory B cell subsets. However, most of the CVID patients with autoimmune features (CVID-AI) have both naïve and multiple memory B cell populations enriched for these inherently autoreactive B cells.

Of particular interest is the expansion of 9G4^+^ B cells in multiple memory populations in CVID-AI, as memory B cells are likely to be effectors of antibody production and autoimmunity. The enrichment of 9G4^+^ B cells in the memory compartment is a common finding among patients with SLE, in which serum 9G4 titers correlate with disease activity (27). Some patients with CVID share other clinical features with SLE, including cytopenias, inflammatory arthritis, and sicca symptoms (4), or specific immune abnormalities, such as expansion of CD19^hi^ B cells (40, 41). Given that loss of tolerance among 9G4^+^ B cells is rarely found in autoimmune conditions other than SLE, our data suggest that the loss of tolerance noted in CVID with autoimmunity may share, at least in part, a similar underlying pathologic mechanism to lupus.

The expansion of 9G4^+^ SW memory B cells in CVID-AI patients strongly supports a breach in the exclusion of autoreactive B cells from germinal centers, a defect that has been well-documented in SLE (10). Figure 2C highlights the failure of effective censoring of autoreactive 9G4^+^ B cells from entering the switched memory B cell compartment in CVID-AI subjects. In contrast, this breach in tolerance was not seen in the CVID-only group, which fits with their lack of autoimmune features. There is emerging evidence that germinal centers in CVID do not form properly (42) resulting in loss of B cell tolerance (34). What drives this defect is unknown, though it may, in part, be driven by the autoimmune cytopenias common in CVID since it is known that proper GC formation is negatively affected by B cell cytopenia (43). Given that 9G4^+^ antibodies bind both erythrocytes and lymphocytes (22, 44) and have been shown to be cytotoxic to B cells (45), it is possible that they perpetuate a destructive cycle of B cell cytopenia, disorganized GC formation, and broken tolerance, leading to further 9G4^+^ B cell maturation and antibody secretion. This destructive cycle could potentially be initiated by autoreactive B cells that escape tolerance and mature via an extra-follicular pathway.

DN2 B cells are theorized to derive from aNAV B cells though a putative extra-follicular pathway (14). Our data show a significant expansion of inherently autoreactive 9G4^+^ B cells in the DN2 population among many CVID patients (Figure 2E). This expansion could be driven by BAFF, which is important for B cell maturation through the extrafollicular pathway (46), and which is elevated in CVID regardless of autoimmune features (47). In contrast, no expansion of 9G4^+^ aNAV B cells was noted. Together, these observations suggest a possible censoring or tolerance checkpoint between the aNAV and DN2 populations that is broken in CVID. These autoreactive B cells that escape tolerance via an extra-follicular pathway might then be able to initiate the destructive cycle noted above. Alternatively, dysfunctional GC formation might occur first, leading to shunting of autoreactive B cells into an extra-follicular pathway with less strict tolerance mechanisms. In any case, and regardless of the initial pathway involved, subsequent B cell autoreactivity might also be induced by circulating 9G4^+^ antibodies themselves, as these antibodies have been shown to induce B cell activation and differentiation (48, 49).

This study highlights the heterogeneity of B cell defects among patients with CVID, including differences among those with autoimmunity. Driessen et al. describe five subsets of CVID patients based on differing defects in B cell development as assessed by replication history and somatic mutation status (6). Using the κ-deleting recombination excision circle assay and the Igκ-restriction enzyme hot-spot mutation assay, they show defects in (1) B cell production, (2) early peripheral development, (3) B cell activation and proliferation, (4) the germinal center reaction, and (5) post-germinal center development. Our data supports this heterogeneity among CVID patients and adds to their findings by focusing on the development and maturation of inherently autoreactive B cells as well as comparing CVID patients with and without autoimmune disease. In addition, we evaluate the more recently characterized DN2 population (14), which plays a significant role in SLE pathogenesis. While significant variability exists within the group, all CVID-AI patients exhibited an expansion of 9G4^+^ B cells in multiple B cell subsets, including the SW memory population, that subset which is similarly dysregulated in SLE and produces the highest affinity autoantibody. In contrast, the CVID group, while also diverse, has no increase in autoreactivity in the memory populations as compared to healthy controls, consistent with their lack of autoimmune features. Broad conclusions based on this study are limited due to the small number of subjects. However, despite these small numbers, consistent patterns and statistically significant differences were noted. Further evaluation of 9G4^+^ B cells and serum antibody in an expanded number of subjects is warranted in order to substantiate these findings. In addition, future studies might address whether this expanded B cell population contributes to autoimmunity via antibody-dependent or -independent mechanisms. In summary, the finding that inherently autoreactive 9G4^+^ B cells are enriched in multiple B cell subpopulations in CVID patients with autoimmune features suggests that this subgroup of CVID patients may have shared B cell tolerance defects with SLE.

## Data Availability Statement

All datasets generated for this study are included in the article/supplementary material.

## Ethics Statement

The studies involving human participants were reviewed and approved by Human Subjects Institutional Review Board, University of Rochester Medical Center. The patients/participants provided their written informed consent to participate in this study.

## Author Contributions

CR and JA contributed to the conception and design of the study. GD, CM, JB, and AP performed the experiments and collected the data. CR, MS, and JA analyzed the data. IS and RL provided the additional interpretation of the data. CR wrote the first draft of the manuscript. MS wrote the sections of the manuscript. All authors contributed to manuscript revision, read, and approved the submitted version.

### Conflict of Interest

The authors declare that the research was conducted in the absence of any commercial or financial relationships that could be construed as a potential conflict of interest.

## Abbreviations

aNAV: activated naïve B cell
CVID: common variable immunodeficiency
CVID-AI: common variable immunodeficiency with autoimmune features
DN: double-negative B cell
DN2: double-negative 2 B cell
GC: germinal center
IgA/G/M: immunoglobulin A/G/M
PBMC: peripheral blood mononuclear cells
SLE: systemic lupus erythematosus
SW memory: class-switched memory B cell
TLR: toll-like receptor
UNSW memory: unswitched memory B cell.
